# Upregulated jasmonate signaling shifts Arabidopsis microbiota interactions and stress adaptations through a positive feedback loop

**DOI:** 10.1093/ismejo/wrag146

**Published:** 2026-06-11

**Authors:** Tung-Tse Lu, Miguelito Isip, Chiao-Jung Han, Hung-Jui Shih, Syukur Syukur, Lai Loi Trinh, Silvina Perin, Yu-Chun Lin, Po-An Lin, Ka-Wai Ma

**Affiliations:** Institute of Plant and Microbial Biology (IPMB), Academia Sinica, Taipei 115201, Taiwan; Institute of Plant and Microbial Biology (IPMB), Academia Sinica, Taipei 115201, Taiwan; Institute of Plant and Microbial Biology (IPMB), Academia Sinica, Taipei 115201, Taiwan; Institute of Plant and Microbial Biology (IPMB), Academia Sinica, Taipei 115201, Taiwan; Institute of Plant and Microbial Biology (IPMB), Academia Sinica, Taipei 115201, Taiwan; Institute of Plant and Microbial Biology (IPMB), Academia Sinica, Taipei 115201, Taiwan; Department of Plant Microbe Interactions, Max Planck Institute for Plant Breeding Research, 50829 Cologne, North Rhine-Westphalia, Germany; Department of Entomology, National Taiwan University, Taipei 106319, Taiwan; Department of Entomology, National Taiwan University, Taipei 106319, Taiwan; Institute of Plant and Microbial Biology (IPMB), Academia Sinica, Taipei 115201, Taiwan; Department of Plant Microbe Interactions, Max Planck Institute for Plant Breeding Research, 50829 Cologne, North Rhine-Westphalia, Germany

**Keywords:** plant microbiota, microbial imbalance/dysbiosis, JA signaling, growth and defense

## Abstract

The model plant *Arabidopsis thaliana* hosts diverse microbial communities collectively known as the microbiota. The plant microbiota is generally taxonomically structured and, in many cases, confers benefits to the plant host including plant growth promotion and enhanced stress tolerance. However, microbial imbalance can also result in deleterious effects, a phenomenon termed dysbiosis that was first coined in the gut microbiome field. To uncover the regulatory mechanism maintaining healthy plant homeostatic interactions with microbiota, we conducted screening using defined synthetic bacterial communities. We identified an Arabidopsis mutant displaying altered microbial profiles with an overall increase of microbial load and microbiota-dependent growth defects. Transcriptomic analyses combined with phytohormone quantification revealed that these phenotypes are attributed to an upregulation of the jasmonic acid (JA) signaling pathway in this mutant upon microbiota colonization. Furthermore, chemical treatment with different JA inducers reproduced similar phenotypes in wild-type plants, suggesting regulation through a positive feedback loop. Although activation of the JA pathway is typically associated with enhanced plant stress responses, our mutant exhibited reduced pathogen load at the expense of reduced plant growth and impaired salt tolerance. Together, our findings demonstrate that JA signaling not only orchestrates plant growth and defense but also plays a pivotal role in shaping plant–microbiota interactions. Controlled regulation of the JA signaling pathway is therefore essential to maintain **balanced plant response** to multiple environmental stressors.

## Graphical Abstract

**Graphical Abstract:**
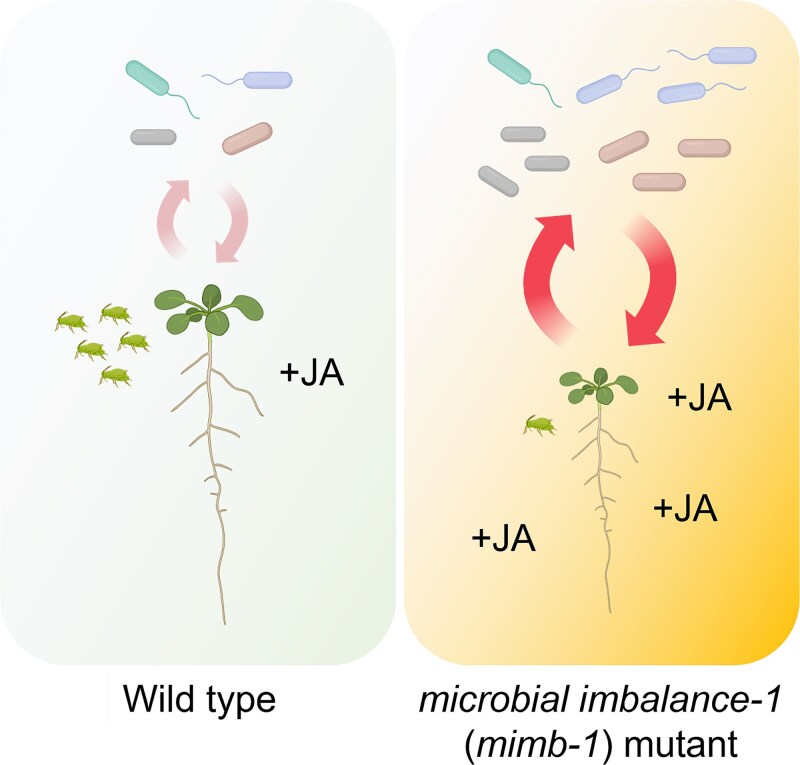

## Introduction

Plants are colonized by a multi-kingdom microbial community, generally known as the plant microbiota [1]. Plant microbiota exhibit multifaceted impacts on their plant hosts. Some microbiota members are deleterious pathogens that exploit plant hosts under favorable conditions. Some are beneficial microbes that provide plant hosts with services ranging from growth promotion to facilitate nutrient uptake or stress resistance [1, 2]. Plant root microbiota such as those of *Arabidopsis thaliana* is dominated by *Proteobacteria* (*Pseudomonadota*), *Actinobacteria* (*Actinomycetota*), and *Bacteroidetes* (*Bacteroidota*) [3, 4]. Reconstitution experiments using synthetic communities comprising core microbiota members of microbial culture collection enables functional characterization of microbiota on plant hosts under controlled laboratory conditions [5–7]. Such a reductionist approach is instrumental in shedding light on the roles of plant innate immunity [8, 9], specialized metabolites chemistry [10–17], and bacterial carbon preference in shaping plant microbiota composition [18]. Although the plant microbiota, especially the bacterial microbiota, is taxonomically structured [19, 20], it is subjected to modulation due to environmental variations or still poorly understood host-mediated regulatory mechanisms. Given that microbiota activities and plant phenotypes are connected, further understanding of the principles governing plant-microbiota establishment can provide means to manipulate microbiota for modulating plant traits such as growth and defense.

The composition of host-associated microbiota sometimes deviates from the “norm”, a phenomenon, i.e. coined “dysbiosis”. Dysbiosis was proposed in the field of the human gut microbiome to describe the microbial imbalance associated with diseases [21, 22]. A major caveat to use this term stems from the implicit causal relationship between microbial imbalance and changes in host phenotypes, which is a classic circular argument that fails to distinguish dysbiosis as a cause, a consequence, or part of a feedback loop. Despite this ambiguity, understanding dysbiosis is crucial to provide a conceptual framework to account for the dynamics of microbiota and their consequences on plant host. Several dysbiosis mutants with known targets or mechanisms were reported in Arabidopsis. For example, mutations in the vesicle trafficking component *min7* and three immune receptors/coreceptors including *fls2 efr cerk1* resulted in phyllosphere microbial imbalance. This quadruple mutant is characterized with a concomitant increase in the families *Comamonadaceae, Xanthomonadaceae, Alcaligenaceae, Sphingomonadaceae,* and a decrease in *Paenibacillaceae* [23]. Mutations in the membrane-bound NADPH oxidase *rbohD* [24], the malectin-like domain-containing receptor *feronia* [25], and the phytosulfokine receptor 1 *pskr1* [26] are also associated with disease-like phenotypes. The disease-like phenotypes were attributed to an opportunistic *Xanthomonas* pathogen for *rbohD* [24, 27, 28] and an overproliferation of *Pseudomonas* species for the others [25, 26]. As such, yet unknown specificities regulated by the corresponding wild-type genes likely exert different impacts on the bacterial microbiota. Consistent with the previous reports showing that plant immunity plays a pivotal role coordinating plant-microbiota homeostasis [8, 9, 29], the dysbiosis mutants aforementioned are immunity-related. A recent genetic screen identified a S-acyltransferase mutant, *tip1,* exhibiting microbiota-dependent autoimmune phenotypes [30]. Together, these studies show a diversity of genes involved in shaping microbiota composition.

We recently showed that individual members of the *A. thaliana* bacterial culture collection [31] exhibited contrasting abilities to modulate root immune responses [8]. Using defined synthetic bacterial communities (SynComs) with taxonomically similar (Table S2) but contrasting traits to activate or suppress root immune responses, we identified plant gene clusters showing differential response to these two groups of bacteria with capacities to activate and suppress root immune responses [8]. We hypothesize that microbiota-mediated differentially expressed gene (DEG) clusters contain candidate genes important for healthy plant–microbiota interactions. To test this idea, we initiated a genetic screen to identify mutants with altered microbial communities, a “bona fide” feature of dysbiosis. We identified an Arabidopsis mutant, *FLAG_264G01*, with the T-DNA disrupting the second exon of a Class III peroxidase gene, *PEROXIDASE 5 (PER5). FLAG_264G01* mutant exhibited phenotypes including microbial overgrowth and microbiota-dependent stunted growth. Based on these data, we renamed *FLAG_264G01* as *microbial imbalance-1 (mimb-1).* However, genetic analysis including the testing of independent CRISPR-edited *PER5* null mutants suggested that mutation of *PER5* alone was not sufficient to cause plant stunting and microbial overgrowth with SynCom. To provide more mechanistic insight, comparative transcriptomic analysis between wild type and *mimb-1* hinted that the jasmonic acid (JA) pathway was upregulated in response to SynCom. JA and the derived methyl ester methyl jasmonate (MeJA), collectively known as jasmonates, are endogenous phytohormone well known for their regulatory role on plant growth and defenses [32]. In Arabidopsis, jasmonates are primarily synthesized via the octadecanoid pathway in the plastids and promote the degradation of the repressor JAZ proteins through the E3 ubiquitin ligase COI1. JA pathway is upregulated in response to both abiotic and biotic stresses [32]. A role of JA signaling in regulating plant–microbiota interactions has been reported. For examples, JA treatment led to community shifts in Arabidopsis [33, 34] and wheat [35]. However, no impact of JA on microbiota was also reported [36]. Some non-pathogenic rhizobacteria were known to activate induced systemic resistance (ISR), which required JA signaling component [37]. Activation of ISR enhanced plant resistance to pathogen and was associated with the recruitment of specific rhizobacteria [38]. In this study, through the use of two JA inducers and microbiota reconstitution experiments, we demonstrated that the upregulation of the JA pathway directly contributed to microbial imbalance phenotypes and established a self-reinforcing feedback loop. Together, an integration between microbiota and proper plant JA signaling exerted a profound impact on plant–microbiota interactions and adaptation to environmental stressors.

## Materials and methods

### Plant materials and growth conditions

*Arabidopsis thaliana* ecotype Columbia (Col-0, CS60000), Wassilewskija (Ws4) were lab stocks. *mimb-1* (FLAG_264G01), *PER4* knock-down mutant (SALK_110617C, *prx4-1*), *PER4* hypermorphic mutant (SALK_044730C, *prx4-2*) [39], and *COAC1* null mutants (SALK_091671C, *coac1-1*; SALK_087365C, *coac1-2*) were obtained from the Nottingham Arabidopsis Stock Center.

*Arabidopsis thaliana* seeds were surface-sterilized according to a published protocol [6]. Briefly, seeds were incubated 5 min each with 70% ethanol twice, followed by a brief wash with 100% ethanol. Seeds were washed thoroughly with sterile water three times and cold-stratified seeds were sowed on agar plates (1%, Difco Bacto Agar, BD Biosciences) supplemented with half-strength Murashige and Skoog (MS) medium (M0222, Duchefa, NL), 0.1 g/L 2-(*N*-morpholino)ethanesulfonic acid (MES, pH 5.7). Plants were grown in a growth chamber under short-day conditions (10 h light, 14 h dark) at 21°C/19°C cycle, 65% relative humidity and light intensity of 120 mE m^−2^ s^−1^ or a controlled growth room. For MeJA and 2,6-dichlorobenzonitrile (DCB) treatment, MeJA and DCB stock solutions were prepared with DMSO and diluted to the indicated concentrations in the MS media (Murashige & Skoog medium including vitamins, Duchefa Biochemie).

### gRNA design and the generation of *PER5* CRISPR lines

Four pairs of gRNA sequences targeting *PER5* gene were designed using the default settings of the ChopChop program [40]. Hybridized gRNAs were inserted into the recipient vector pDEG347 though shuttle vectors using the published restriction-ligation protocol [41]. Same construct was used to transform Arabidopsis plants in both Col-0 and Ws4 background. Arabidopsis was transformed by *Agrobacterium tumefaciens* GV3101 pMP90 carrying the final transformation vector using the floral dipping method. Briefly, inflorescences of plants were dipped into an *Agrobacterium* solution (an LB-grown saturated bacterial culture resuspended in 5% sucrose solution and 0.02% Silwet L-77). Primary transformants were selected by resistance to the herbicide phosphinothricin. Oligonucleotides used in this study can be found in Table S1.

### Culture conditions for bacteria

Culture collection members, *A. tumefaciens* GV3101 and *Pseudomonas syringae* (*Pto*DC3000) were grown on 50% tryptic soy broth agar plate (Sigma–Aldrich, USA or cyrusbioscience, Taiwan), Luria-Bertani broth or King’s B medium (HiMedia, USA) at 25°C, 25°C, and 28°C from one to four days, respectively. For the preparation of SynCom, fully grown bacterial cultures were pelleted by centrifugation at 8000 g for 5 min, followed by two washes and resuspension in 10 mM MgSO_4_. Strains were inoculated to warm agar medium at a concentration of OD_600_ = 0.0005 for each strain. Two 16-member SynComs (*At-*16SC1 and *At*-16SC2) were prepared. The composition of *At*-16SC1 is similar to the previously published *At*-SC4 [42] except for the replacement of strain root1221 with stain29. *At*-16SC2 is similar to *At*-16SC1 except for the replacement of strain R418 by R336D2 (both of them are *Oxalobacteraceae* strains) due to the loss of R418. The composition of the SynCom could be found in Supplementary Table S2.

### Reinoculation experiment

Ws4 and *mimb-1* plants were grown from seeds with *At-*16SC1 for 2 weeks. Ws4 and *mimb-1* seedlings were harvested and homogenized in 10 mM MgSO_4_. The inocula were diluted to final concentrations of 0.01 g tissue/ml and 0.001 g tissue/ml for Ws4 and *mimb-1*, respectively. This dilution was needed to make the bacterial titer comparable across the two genotypes. Bacterial titers were validated by plate counting method. The plant extracts were mixed with full strength MS agar to obtain half strength MS medium (final concentration of MES and agar remain unchanged).

### DAB staining

Seven-day Ws4 or *mimb-1* seedlings with or without *Pseudomonas* strain root9 were stained with 1 mg/ml DAB prepared in a 10 mM phosphate buffer (pH 7.0) for 1 h. After three washes, the seedlings were observed under a stereomicroscope. The intensity of DAB at the root tip was quantified by ImageJ. The raw images were converted to the RBG mode with a fixed threshold set as 70. Rectangle tool was used to select a fixed area as the region of interest covering the root tip. The area normalized signal density (mean) was quantified. The mean values were subtracted from the background (set as 255) for subsequent analyses.

### Quantification of jasmonic acid and salicylic acid levels

19 days old Ws4 and *mimb-1* plants were germinated on half strength MS agar plate supplemented with SynCom *At*-16SC1. Plants are separated from the growth media by a 100-micron nylon mesh (Nitex Cat# 03-100/44, Sefar) to minimize the carrying over of nutrient from the agar matrix that will interfere with the metabolomics analyses. Roots and shoots were harvested separately. Thirty milligrams of tissues were pulverized (Tissuelyser II, Qiagen). Metabolites were extracted using an extraction buffer containing a 40:40:20 volume ratio of acetonitrile (ACN): methanol: 0.06% hydrochloric acid + 20 ng/ml salicylic d4 as an internal standard. Briefly, 1 ml pre-cooled (−20°C) extraction buffer was added to each sample, followed by incubation at 1500 rpm in a thermomixer for 30 min at 4°C. Samples were centrifuged at 21 000 g for 10 min. The pellets were used to determine protein concentrations using a bicinchoninic acid assay. The cleared supernatants were transferred to a new tube and dried in a Speed Vac concentrator (Centrivap, Labcono) at 10°C at 1000 rpm. Dried samples were re-suspended in 100 μl solution containing 50:50 volume ratio of ACN (LC–MS grade) and 10 mM ammonium formate supplemented with 0.15% formic acid (FA). Samples were vortexed for 30 s and centrifuged for 2 min at 10 000 g and 4°C. The cleared supernatants were transferred to 200 μl glass inserts (CZT; Germany). All samples were placed in an Acquity iClass UPLC (Waters) sample manager held at 10°C. The UPLC was connected to an Orbitrap, equipped with a heated ESI (HESI) source (QExactive, Thermo Fisher Scientific). Two microliters was injected onto a 100 × 1.0 mm HSS T3 C18 UPLC column, packed with 1.8 μm particles (Waters). The flow rate of the UPLC was set to 100 μl min^−1^ and the buffer system consisted of buffer A (10 mM ammonium formate and 0.15% FA in UPLC-grade water) and buffer B (UPLC-grade ACN). The UPLC gradient was as follows: 0–1 min 90% A, 1–8 min 90%–10% A, 8–10 min 10% A, 10.01–12 min 90% A. This leads to a total runtime of 12 min per sample.

The QExactive mass spectrometer was operating in negative ionization mode scanning a mass range between m/z 50 and 750. The maximal ion time was set to 200 ms and the HESI source was operating with a spray voltage of 2.75 kV in negative ionization mode. The ion tube transfer capillary temperature was 350°C, the sheath gas flow 50 arbitrary units (AU), the auxiliary gas flow 14 AU, and the sweep gas flow was set to 3 AU at 380°C. All samples were analyzed in a randomized run order. Targeted data analysis was performed using the Quan module of the TraceFinder 4.1 software (Thermo Fisher Scientific) in combination with a sample-specific compound database, derived from measurements of commercial reference compounds (Sigma). Samples below the detection limit were not excluded from the analyses. Instead, these samples were replaced by half of the lowest detection values (DL/2).

### 16S ribosomal RNA gene amplicon sequencing and community profiling

Libraries were processed according to previously published protocol [8]. Briefly, plant roots and shoots were harvested separately unless otherwise specified. Total DNA was extracted using the FastDNA SPIN Kit for Soil (MP Biomedicals) and the ZymoBIOMICS DNA Kit (Zymo Research) according to the manufacturer’s instructions. Samples were diluted to 3.5 ng/μl before being used as templates in a three-step protocol [8] or a modified one-step PCR protocol. The V5V7 region of the bacterial 16S ribosomal RNA (rRNA) gene was amplified using the primer pairs 799F and 1192R (Table S1). Spike-in DNA [43] was added during the first PCR amplification to enable normalization of the data. We prepared a spike in plasmid as a 0.03 ng/μl stock [43]. 0.075 μl spike-in DNA plasmid (or 2.25 pg) was added in the master mix for each sample (25 μl reaction each in triplicates +25 μl as a negative control, total 75 μl) during the first PCR to enable normalization of the data. Forward and reverse sequencing reads were denoised and demultiplexed separately according to the barcode sequences using QIIME [44] with a barcode error threshold of 0.1. After quality-filtering, merging of paired-end reads into amplicon tags using FLASH2, ambiguous bases were filtered out by *fastq_filter* command in USEARCH [45]. One out of three biological replicates shown in the first 16S rRNA gene sequencing was not analyzed due to contamination with a *Xanthomonadales* strain for the reconstitution experiment. In total, 10 replicates collected from two biological repetition were analyzed for each treatment. Correction of PCR and sequencing errors was performed using the Rbec pipeline [46]. An Rbec-derived amplicon sequence variant (ASV) table with 100% identity to the references for each strain was generated. The taxonomies of the SynCom members were classified based on whole genome sequences using Taxator-tk [47]. To account for variation in sequencing depth, ASV counts were normalized to spike-in counts by calculating the ratio of Rbec-derived ASVs to the total spike-in reads per sample.


\begin{align*} &\mathrm{Spike}-\mathrm{in}\ \mathrm{normalized}\ \mathrm{ASV}\ \mathrm{abundance}\\&\qquad=\left(\mathrm{Rbec}-\mathrm{derived}\ \mathrm{ASV}\ \mathrm{read}\ \mathrm{counts}/\mathrm{Spike}-\mathrm{in}\ \mathrm{read}\ \mathrm{counts}\right) \end{align*}

This ASV table was used for subsequent diversity analyses. Bray–Curtis dissimilarity matrix was calculated using the *vegdist* function in the vegan package [48]. Constrained PcoA was performed using the vegan *capscale* function on the Bray–Curtis dissimilarity matrices, constraining by the following formula.


\begin{align*} & {\mathrm{genotype}}^{\ast }\ \mathrm{compartment}\\&\qquad\qquad+\mathrm{Condition}\ \left(\mathrm{biological}.{\mathrm{replicate}}^{\ast }\ \mathrm{technical}.\mathrm{replicate}\right) \end{align*}

Differential abundance analysis was performed using *limma* [49]; data were log2-transformed, followed by linear modeling and empirical Bayes moderation to stabilize variance. Significance was determined via moderated t-tests with a false discovery rate correction for multiple testing. Log_2_ fold change (LFC) scatter plots and bubble plots were generated using the *phyloseq* package [50] to integrate the ASV table, taxonomy, and experimental factors (genotype, treatment, and genotype_treatment groups). Pairwise Wilcoxon rank-sum tests in the bubble plot using the *wilcox.test* function in basic R were performed within each compartment to compare experimental groups against the wild type (Ws4) mock reference. *P* values were adjusted for multiple testing using the Benjamini–Hochberg (BH) correction using *stats* [51]. Microbial community structure was evaluated using ANOSIM, Adonis2 (PERMANOVA), and PERMDISP (betadisper) within the *vegan* package [48]. All tests were based on Bray–Curtis dissimilarity matrices. Pairwise comparisons for each method were conducted using the *pairwiseAdonis* package [52] and *vegan* [48]. Mean Bray–Curtis dissimilarity within and between groups was calculated using the mean function in *dplyr* [53]. All amplicon data were visualized using the *ggplot2* [54].

### Stressor experiments: *Pseudomonas* pathogen growth and salt tolerance

For resistance against bacterial pathogens, Ws4 and *mimb-1* plants were grown in potting soil for 4–5 weeks. Leaves were infiltrated with a bacterial suspension *of Pseudomonas* strain *PtoDC3000/Rif* (O.D._600_ = 0.0001, Animal and Plant Health Inspection Agency import permit number: **112-B-510**) in 10 mM MgSO_4_. Plants were covered to maintain high humidity. Infiltrated leaves were harvested after 3–4 days before notable symptoms appeared. The pathogen titer per leaf disc (8 mm diameter) was quantified through a viable plate counting method after serial dilution. We also performed pathogen infection experiment under axenic and SynCom inoculated conditions in the agar plates. Plants were grown in axenic or SynCom-inoculated square plates for two weeks followed by flooding inoculation with *PtoDC3000/Rif* (O.D._600_ = 0.0001). The pathogen suspension solution was removed and the plates were placed back to growth chamber for 3 days. For salt tolerance, plants were germinated on agar plates under axenic and SynCom-inoculated conditions for 7 days before being transferred to freshly prepared agar plates with the indicated concentrations of NaCl. Shoot fresh weights were measured at Day 17 or 10 days after transfer.

### Herbivore resistance and preference evaluation

Arabidopsis seedlings were grown for 3 weeks before the experiments. *Myzus persicae* (wingless adult aphids) and *Spodoptera litura* (second instar larvae caterpillars) were used as representatives of pierce-damaging and chewing-damaging herbivores, respectively. One hundred sixty aphids and eighty caterpillars were used in each batch of experiments. They were placed on a paper bridge with connections to both wild-type Ws4 and *mimb-1* mutant. Insects with and without selection were scored after 6 min. Proportion of insects showing a preference and the corresponding selection rate for each genotype was calculated. The free choice experiments were repeated for a total four times. Statistical analyses were performed based on beta regression test.

To eliminate the variation of size on the insect preference between Ws4 and *mimb-1*, we performed non-choice assay and evaluated the fitness of both herbivores. Aphid was evaluated by total nymphs produced after feeding with the corresponding plant materials. Caterpillar was evaluated by caterpillar weight gain and the efficiency of conversion of ingested food (ECI). Three aphids were placed on either axenic or 16-member SynCom inoculated Ws4 or *mimb-1*. The total nymphs were calculated after 5 days. In caterpillar assay, caterpillars were feed with two different plant lines separately. The weights of the caterpillars were measured before and after the experiment. The input plant weight and the remaining weight were also recorded. The ECI value is calculated by (weight gain of caterpillar/ caterpillar consumed plant weight)*100%. Higher ECI value indicated higher fitness gain upon feeding on one plant line. The non-choice experiment was repeated a total of three times, each time having five replicates consisting of three individuals each.

### Transcriptome experiments

Plants were germinated with the *At-*16SC1 for 14 days before harvesting using the same procedure of inoculation as described in the section culture conditions for bacteria. Roots and shoots were harvested separately and froze in liquid nitrogen immediately. RNA from nine different samples collected from three biological replicates were extracted with the Plant RNeasy Mini Kit (Qiagen) according to the manufacturer’s instructions. After a quality check, libraries were prepared by Novogene-Europe and sequenced on a NovaSeq 6000 System (Illumina) each with a minimum 3 Gb sequencing depth. Across samples, between 9.8 and 14.4 million raw reads were obtained, with 9.1–13.1 million reads successfully mapped, corresponding to alignment rates of 90.8%–95.5% (Table S3).

Raw reads were preprocessed using *fastp* [55] with default settings for paired-end. High quality reads were pseudo-aligned to TAIR10.58 *A. thaliana* transcriptome reference (Ensembl) [56] using kallisto [57]. On average, we obtained 11.61 million paired-end reads per sample. After removal of low abundant transcripts that were absent in at least two replicates under each condition, count data were imported using the *tximport* package [58]. The log_2_ scaled counts were normalized by the identified SVs [59] using the *limma* package (*removeBatchEffect* function) [49] and transformed as median-centered *z*-score by transcripts (scaled counts, “scale” function). Then *z*-scores were used to conduct *k*-means clustering for all transcripts. The cluster number (*k* = 8) was determined by sum of squared error and Akaike information criterion.

Differential expression analyses were performed using the *DESeq2* package [60]. Pairwise comparisons were designed as: (i) *mimb-1* mock vs WT mock, (ii) SynCom-treated WT vs WT mock, (iii) SynCom-treated *mimb-1* vs SynCom-treated WT, (iv) SynCom-treated *mimb-1* vs *mimb-1* mock. Transcripts with fold-changes >1.5 with adjusted *P* value equal to or below 0.05 were considered significant.

Gene ontology (GO) enrichment for each cluster using the whole Arabidopsis transcriptome as background were performed with the *GOseq* package [61] with the consideration of transcript length. GO annotations were retrieved from the Gene Ontology Consortium (February 2025). Significantly changed biological process GO terms (adjusted *P* value <.05) were visualized in dot plots using the *clusterProfiler* package [62].

### Genomic analyses searching for jasmonic acid-metabolic genes in selected microbiota members

To identify bacterial homologous genes involved in JA biosynthesis, genomes of the individual members from the 16-member SynCom were used as the queries. Protein sequences were aligned against the references in Arabidopsis downloaded from UniPort by DIAMOND (version 2.1.11) using the ultrasensitive mode [63]. Genes showing more than 35% sequence identity were used in the subsequent analyses and visualized based on customized scripts. Bacterial phylogeny of these 16 members was constructed using the Type (Strain) Genome Server [64] based on whole-genome sequences.

### Statistical analysis

All experiments were performed in total of three times (*n* = 3) unless otherwise specified. Analyses were performed using the R environment. Dunn’s Kruskal–Wallis and ANOVA were used to test for statistical significance. A *P* value smaller than 0.05 was considered significant unless otherwise specified.

## Results

### Identification of a mutant with microbiota-dependent stunted growth

**Figure 1:**
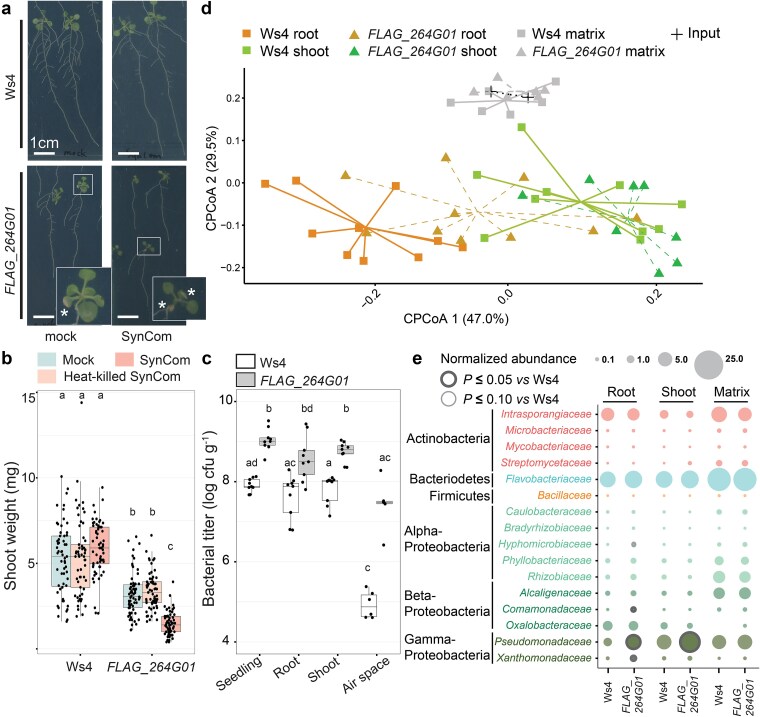
Identification of *FLAG_264G01* mutant with microbial imbalance phenotypes using SynComs. Wild-type Ws4 and *FLAG_264G01* were grown for 2 weeks on agar matrix in the presence of the 16-member SynCom *At*-16SC1. (a) Representative images of Ws4 and *FLAG_264G01* grown with *At*-16SC1. The inserts corresponded to enlarged images of individual plants with asterisks indicating leaves showing chlorosis-like phenotype. (b) Fresh shoot weight of plants treated with live or heat-killed SynCom. (c) Quantification of bacterial load in different compartments by live colony counts. (d) Constrained PCoA plot of Bray–Curtis dissimilarities (ASV level) constrained by replicates. Shapes represent genotypes and colors represent compartments. Initial input inocula were labelled as input. (e) Balloon plot showing the spike normalized abundance of individual families colonizing the indicated compartments and genotypes. Statistical significances were determined by Kruskal–Wallis followed by Dunn’s post-hoc test in (b) & (c), PERMANOVA in (d), and pairwise Wilcoxon rank-sum test in (e). Different letters or symbols indicated statistical significance of *P* ≤ 0.05 unless otherwise specified.

To identify microbial imbalance mutants, we performed reverse genetic screens using selected Arabidopsis T-DNA insertion mutants and a 16-member bacterial synthetic community SynCom (*At-*16SC1). The 16-member SynCom comprised members from 16 different bacterial families, representing a diversity of strains that were previously shown to exhibit robust colonization on Arabidopsis [42]. We prioritize our screening on mutants of genes with differential transcriptomic responses to microbiota of varying immunomodulatory traits [8]. By focusing on mutants exhibiting microbiota-dependent phenotypes 2 weeks after inoculation compared to wild-type parent (WT), we identified a mutant with significant stunted plant growth in the presence of SynComs (Fig. 1a). Based on the FLAGdb/FST database [65], this mutant (*FLAG_264G01*) carries a T-DNA insertion at the second exon of the *PEROXIDASE 5* gene (*PER5*) in the Wassilewskija-4 (Ws-4) accession background. Peroxidases were involved in ROS production and *PER5* was reported as a defense marker gene [66]. However, an implication of *PER5* for interactions with the microbiota was not known.

Under agar-grown axenic conditions with replete nutrients, *FLAG_264G01* mutant exhibited relatively mild growth defects with 15%–20% less fresh weight compared to the wild-type parent Ws-4 (Fig. 1b, Supp Fig. S1). The presence of SynCom resulted in up to 80% reduction of fresh weight for *FLAG_264G01* compared to the axenic control (Fig. 1b, Supp Fig. S1A–B). In contrast, inoculation with a heat-killed SynCom did not result in stunted growth (Fig. 1b). *FLAG_264G01* showed more stunted growth when grown on agar plate compared to those grown in both potting and natural soils (Fig. 1a, Supp Fig. S1A), suggesting that the stunted growth phenotype is possibly affected by the growth environment. To investigate whether microbiota-mediated growth inhibition in *FLAG_264G01* is dependent on the capacities of microbiota to activate or suppress immunity [8], we tested two additional SynComs: a five-member immune non-suppressive SynCom (NS1) and a five-member immune-suppressive SynCom (S1) [8]. Inoculation of *FLAG_264G01* with these SynComs resulted in comparable growth inhibition to *At*-16SC1 (Fig. 1b, Supp Fig. S1B). Treatment of soil-grown *FLAG_264G01* (presumably already stunted due to the presence of the native microbiota) with *At*-16SC2 did not result in further growth inhibition (Supp Fig. S1D). This result together with our observations that growth inhibition phenotypes were reproducible using three different SynComs, suggested that microbiota-mediated growth inhibition was general. We occasionally observed chlorosis-like phenotypes on *FLAG_264G01* leaves after SynCom treatment (Fig. 1b, one-third of our experiments), but the underlying cause is unclear.

### *FLAG_264G01* growth phenotype was associated with microbial overgrowth and community shift

The growth defect of *FLAG_264G01* was associated with microbial overgrowth. Specifically, *FLAG_264G01* hosted approximately 10-fold higher total bacterial load compared to WT upon inoculation with the tested SynComs (Fig. 1c, Supp Fig. S1C). Unlike previously reported dysbiosis mutant that exhibited compartment-specific effect [23], microbial overgrowth was noted in both roots and shoots as well as the leaf endo-compartment, indicative of an overall change of microbiota composition (Fig. 1c).

We performed community profiling using partial sequences of the V5–V7 region of the 16S rRNA gene. We spiked in a known amount of exogenous DNA during library preparation to enable normalization of the total microbial load. In line with our hypothesis that *FLAG_264G01* is a microbial imbalance mutant, the microbial community of *FLAG_264G01* significantly differed from WT (root: R^2^ = 0.33, *P* < 0.05, shoot, R^2^ = 0.18, *P* < 0.05, matrix: R^2^ = 0.01, *P* = .0.148, PERMANOVA, Table S3). The community shift was observed in the plant-associated compartments (i.e. shoot and root, but not in the agar matrix, Fig. 1d). *Proteobacteria* families including *Pseudomonadaceae, Xanthomonadaceae*, and *Comamonadaceae* were significantly enriched in *FLAG_264G01* roots compared to WT (Fig. 1d, pairwise Wilcoxon rank-sum tests, *P* < 0.05). Among them, *Pseudomonadaceae* exhibited the highest enrichment from 1.23% to 4.54% (Fig. 1e). Based on these results, we renamed this mutant as a *microbial imbalance mutant* (*mimb-1*) hereafter.

**Figure 2:**
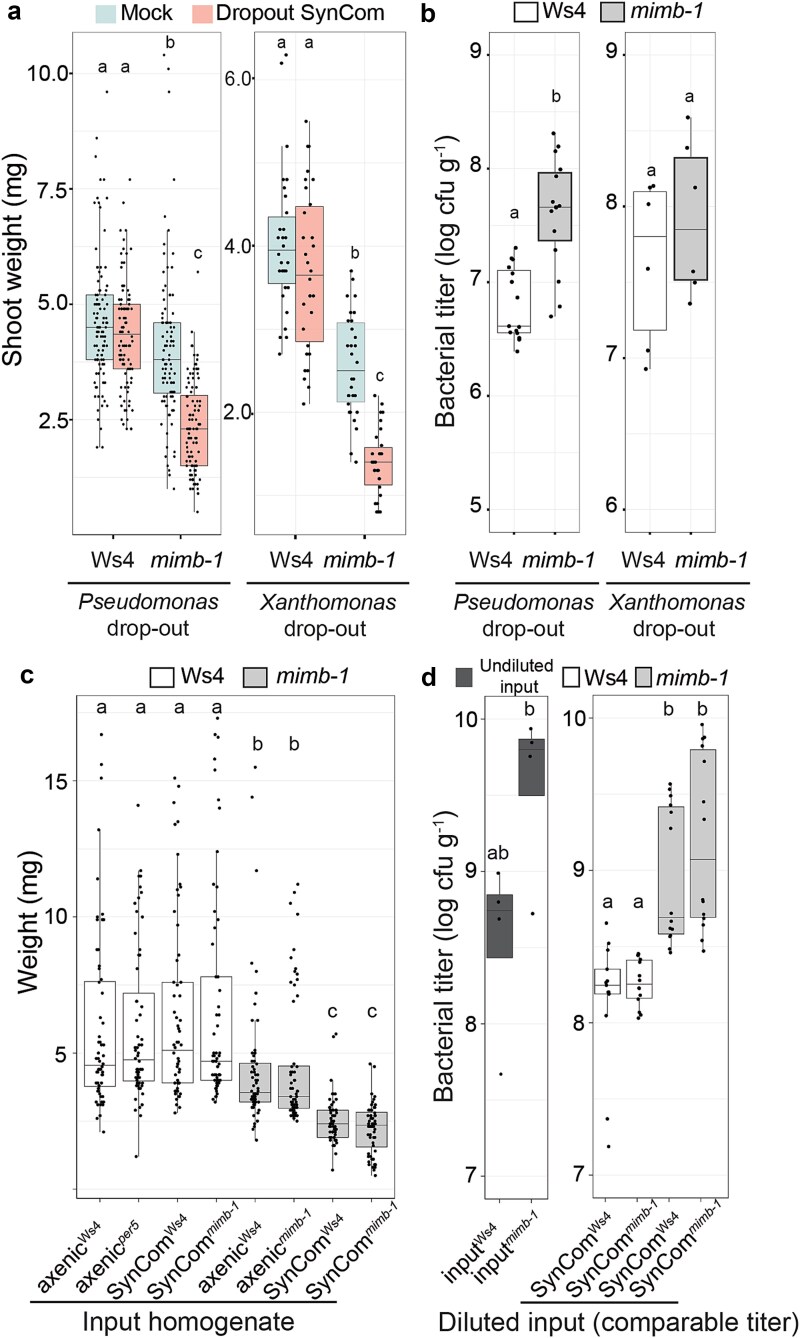
*mimb-1* exhibited features that were atypical for dysbiosis mutants. Plants were grown for 2 weeks on agar matrix in the presence of the 15-member dropout SynCom. Two drop-out SynComs have the same composition of the *At*-16SC1 except for the removal of the *Pseudomonas* strain R68 and *Xanthomonas* strain R480. (a) & (b) Shoot weight and total microbial load of plants treated with the respective 15-member dropout SynCom. (c) & (d) Shoot weight and total microbial load of plants inoculated with axenic Ws4 or *mimb-1* homogenate (axenic^Ws4^ and axenic*^mimb-1^*) or the SynCom-inoculated homogenate (SynCom^Ws4^ and SynCom*^mimb-1^*). Statistical significance was determined by Kruskal–Wallis followed by Dunn’s *post hoc* test. Different letters indicated statistical significance of *P* ≤ 0.05 unless otherwise specified.

We then performed two drop-out experiments to remove the *Pseudomonas* strain or the *Xanthomonas* strain from the 16-member SynCom. The resultant 15-member drop-out SynCom still induced significant growth inhibition on *mimb-1* but not WT (Fig. 2a). We also observed a significant increase in the total microbial load of *mimb-1* compared to WT with the *Pseudomonas* dropout SynCom (Fig. 2b). We did not observe any significant difference with the *Xanthomonas* dropout SynCom. Overall, these results suggested that the deleterious effect from our 16-member SynCom is not due to the over proliferation of one strain alone.

### *mimb-1* exhibited atypical feature compared to known dysbiosis mutant

Following the definition of dysbiosis proposed by the human gut microbiome research, true dysbiosis should fulfil causality between specific microbiota composition and changes in host phenotypes. To address the role of microbial imbalance on plant performance, we prepared WT or *mimb-1* derived homogenate carrying the respective microbial communities as starting inocula, followed by inoculation on WT or *mimb-1* plants. The plant growth phenotypes from these re-inoculation experiments exhibited variation, possibly due to the interference by co-extracted plant factors including nutrients and differences in microbiota extraction efficiency. However, we showed that *mimb-1* derived microbes did not recapitulate microbiota-mediated plant growth inhibition and microbial overgrowth in wild-type Ws4 plants (Fig. 2c and d). Because *mimb-1* accommodated 10 times more microbes compared to WT (Fig. 2d). Accordingly, we used diluted *mimb-1* derived homogenates to make the input titers comparable. Therefore, we concluded that the microbial composition from *mimb-1 per se* did not lead to dysbiosis. Together, we identified a microbial imbalance mutant with microbiota-dependent stunted growth, shift in microbiota composition, and enhanced microbial load.

### *PER5* gene loss-of-function in *mimb-1* was not sufficient to cause dysbiosis

*FLAG_264G01* (or *mimb-1*) was annotated to have a T-DNA insertion at the second exon of the class III peroxidase gene *PER5* (Supp S2A). There are 73 Class III peroxidases in *A. thaliana*, implicated in diverse functions ranging from plant development, abiotic and biotic stress responses [67]. Consistent with a putative role of PER5 in modulating reactive oxygen species (ROS) level, *FLAG_264G01* mutant exhibited reduced basal and microbe-induced ROS by DAB staining (Supp S2B). Although *PER5* carried a T-DNA insertion at the exon, expression analyses using primers targeting both upstream (R1) and two downstream regions (R2 and R3) of the *PER5* T-DNA insertion site showed that *PER5* transcripts were expressed (Supp S2C). To unambiguously investigate the contribution of *PER5* loss-of-function to the dysbiosis-like phenotypes, we generated two independent CRISPR deletion mutants of *PER5, per5 #3-1* and *per5 #5-2* in the Col-0 background (Supp S2D). None of them showed any microbiota-dependent stunted growth (Supp S2E).

We expanded our analyses on the genes surrounding the *PER5* locus. The expressions of two neighboring genes within 4 kb proximity of the *PER5* coding regions, another peroxidase gene *PEROXIDASE 4 (PER4)* and the mitochondrial CoA transporter (*CoAC1)*, were also affected. To assess the contribution of these two genes, *PER4* and *CoAC1,* to the phenotypes, we tested a knockdown mutant of *per4* (*prx4-1*), a hypermorphic mutant of *PER4* (*prx4-2*) [39] and two null mutants of *CoAC1 (coac1-1* and *coac1-2)*. None of them showed any microbiota-dependent stunted growth (Supp S2E). Therefore, *PER5* loss-of-function alone was not sufficient to cause stunted plant growth phenotypes with SynCom. However, we cannot exclude the possibility that the phenotypes were contributed by unknown epistatic interactions involving multiple genes or still uncharacterized polymorphisms in the *FLAG_264G01* background. To investigate the contribution of background variation (e.g. between Col-0 and Ws4) on our phenotypes, we generated another *PER5* deletion mutant in the Ws4 background (Supp Fig. S2F). This *per5* deletion mutant did not show any significant stunted plant growth (Supp Fig. S2F) or any significant increase in total microbial load with SynCom (Supp Fig. S2G) despite limited number of replicates available. Together, we concluded that neither *PER5* loss-of-function or the genetic background alone fully explained our underlying phenotypes. The genetic factor(s) contributing to microbial imbalance will require further investigation.

### Jasmonic acid pathway was upregulated in the *mimb-1* mutant

**Figure 3:**
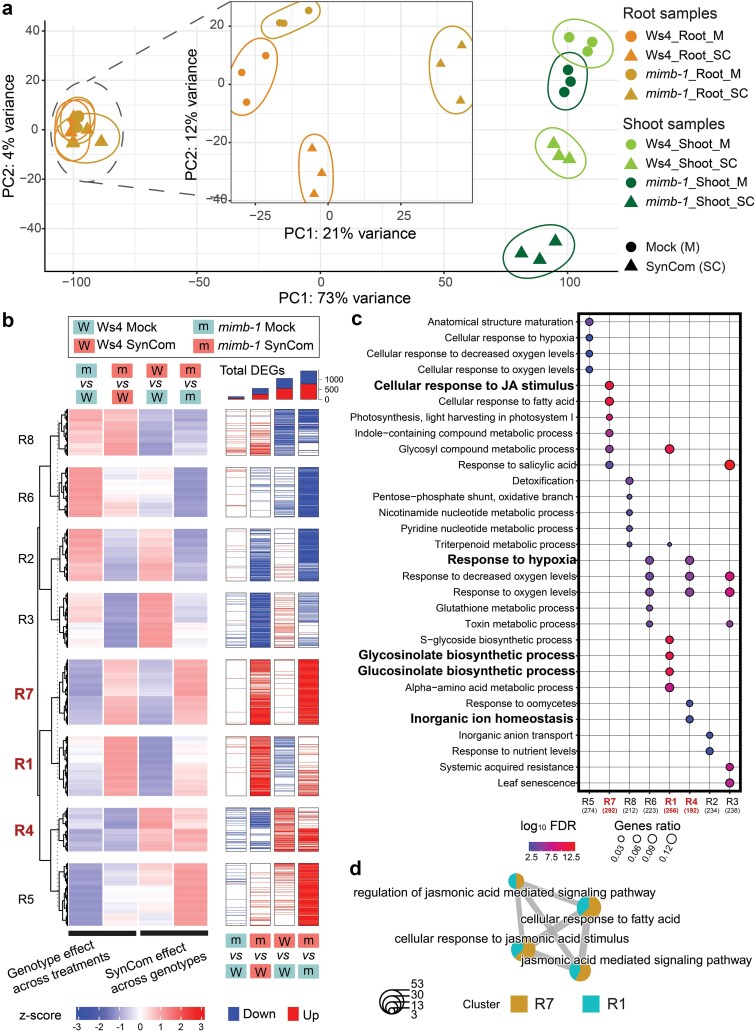
*mimb-1* had distinct transcriptomic responses to the synthetic bacterial community. Plants were grown for 2 weeks on agar matrix in the presence of the SynCom *At*-16SC1. Root and shoot samples were harvested separately and subjected to RNA-seq analysis. (a) Principal component analyses showing the separation of transcriptomes in different samples. The insert corresponded to the result using root-associated samples only. Shapes corresponded to mock and SynCom treatment. Colors represented compartments. (b) Left panel, heatmaps summarizing the expressions of genes across genotypes in roots. Right panel, DEGs calculated by pairwise comparisons (log_2_FC > 1.5, *P*adj. < 0.05). For example, a red line upon pairwise comparison between *mimb-1* mock vs Ws4 mock (vertically arranged) indicated that the gene was significantly upregulated in *mimb-1* mock treatment. (c) Top five significantly enriched GO terms associated with each cluster. (d) GO terms related to JA processes and their association with clusters R1 and R7 in the roots. Size of pie charts corresponded to the number of DEGs.

Although the genetic determinant(s) contributing to microbial imbalance remained elusive, we sought to identify biological processes contributing to our phenotypes by performing transcriptomic analyses of WT and *mimb-1* plants in the presence or absence of the 16-member SynCom (Table S4). Using principal component analysis (PCA), the first PC accounted for 73% of the variation, separating root and shoot samples. The second PC separated axenic from SynCom-treated plants (4% variation). Although plant genotype (e.g. Ws4 vs *mimb-1*) only explained a minority of the variation, *mimb-1* still formed distinct clusters from WT under both axenic and SynCom-inoculated conditions in both roots and shoots (Fig. 3a). The bigger separation of transcriptomes in the presence of SynCom compared to axenic plants was consistent with the plant phenotypes (e.g. more stunted growth in the presence of SynCom, Fig. 1a and b).

To better visualize the genotype-specific differences, we performed hierarchical clustering separately on z-score transformed root and shoot dataset. We identified sets of DEGs (log_2_FC > 1.5, *P* < 0.05) using pairwise comparisons (Fig. 3b, Tables S5–S6), followed by GO term enrichment analysis (Fig. 3b, Supp Fig. S3, Table S7). As such, we grouped our dataset into eight clusters. From our root dataset, we identified a SynCom-responsive cluster, **R4**, which was upregulated across both genotypes (Fig. 3c). GO term enrichment analyses suggested that cluster **R4** was linked with hypoxia and inorganic ion homeostasis. In line with previous report [8], these biological processes represented GO terms generally responsive to bacterial SynComs. We identified another SynCom-responsive cluster, **R1**, exhibiting contrasting responses across genotypes (e.g. upregulated in *mimb-1* but not in WT). **R1** genes were associated with functions related to glucosinolate biosynthesis and regulation of the JA signaling pathway (Fig. 3c and d, Table S7). We also identified cluster **R7**, another SynCom-responsive cluster, which was upregulated in *mimb-1* but not WT. **R7** was enriched with genes related to defense, glucosinolate specialized metabolism, and JA responses (Fig. 3c and d, Table S7). We then focus on the JA pathway for further analyses.

**Figure 4:**
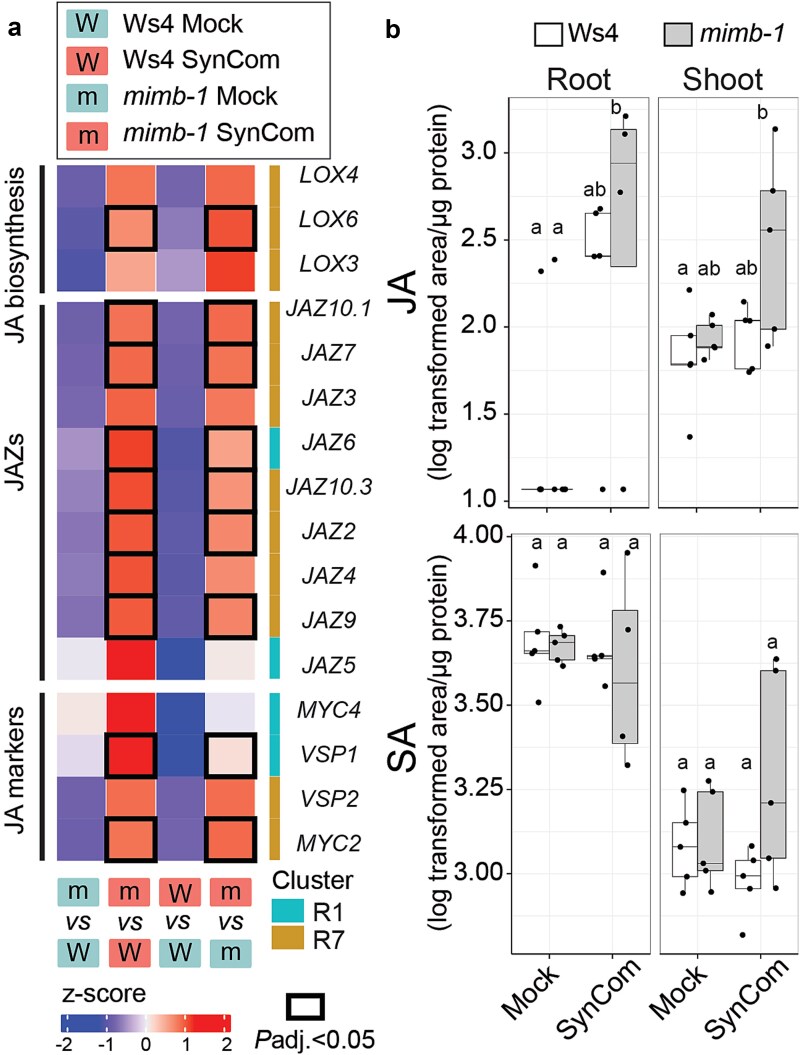
JA levels and related genes were upregulated in *mimb-1* in response to the synthetic community. (a) Normalized relative expression levels of selected genes involved in JA-related processes in roots (related to Fig. 3b). Genes of statistical significance (log_2_FC > 1.5, *P*adj. < 0.05) were indicated with blocked lines. (b) The amount of JA and SA of 2 weeks old Ws4 and *mimb-1* plants in the presence or absence of SynCom *At*–16SC1. Statistical significance was determined by Kruskal–Wallis followed by Dunn’s *post hoc* test. Different letters indicated statistical significance of *P* ≤ 0.05 unless otherwise specified.

To investigate whether JA-related processes were affected at the biosynthesis or the signaling level, we grouped our data and quantified the expression levels of genes involved in JA biosynthesis (e.g. *LOX3, LOX4*), regulation (e.g. multiple *JAZs*), and key transcription factors/markers (e.g. *PDF1.2, MYC2, VSP1*) based on our RNA-seq dataset. All of them were upregulated in *mimb-1* compared to WT in the presence of SynCom (Fig. 4a and b, Supp Fig. S4, Table S8), suggesting that the JA signaling pathway was upregulated. Multiple *JAZs* were also upregulated. JAZs are repressor proteins that negatively regulate JA signaling [32]. Previous study showed that JAZs were upregulated due to JA regulation through a negative feedback mechanism [68–70]. We thus speculated that the upregulation of JAZs was the result of negative feedback in response to hyperactivation of the JA pathway in the *mimb-1* background.

To understand the dynamics of JA in response to bacterial SynCom, we also quantified the levels of JA across both genotypes. We did not detect any JA in roots under axenic conditions. However, inoculation with SynCom *At-*16SC1 resulted in elevated levels of JA. The increase was significant only in *mimb-1* but not WT (Fig. 4b, Supp Fig. S5). In contrast, we observed an increase in another phytohormone such as salicylic acid (SA), but it was not significant. Together, our data suggested that the upregulation of JA cannot be explained by antagonism between JA and SA pathway.

### Manipulation of the jasmonic acid signaling pathway altered plant microbiota interactions

**Figure 5:**
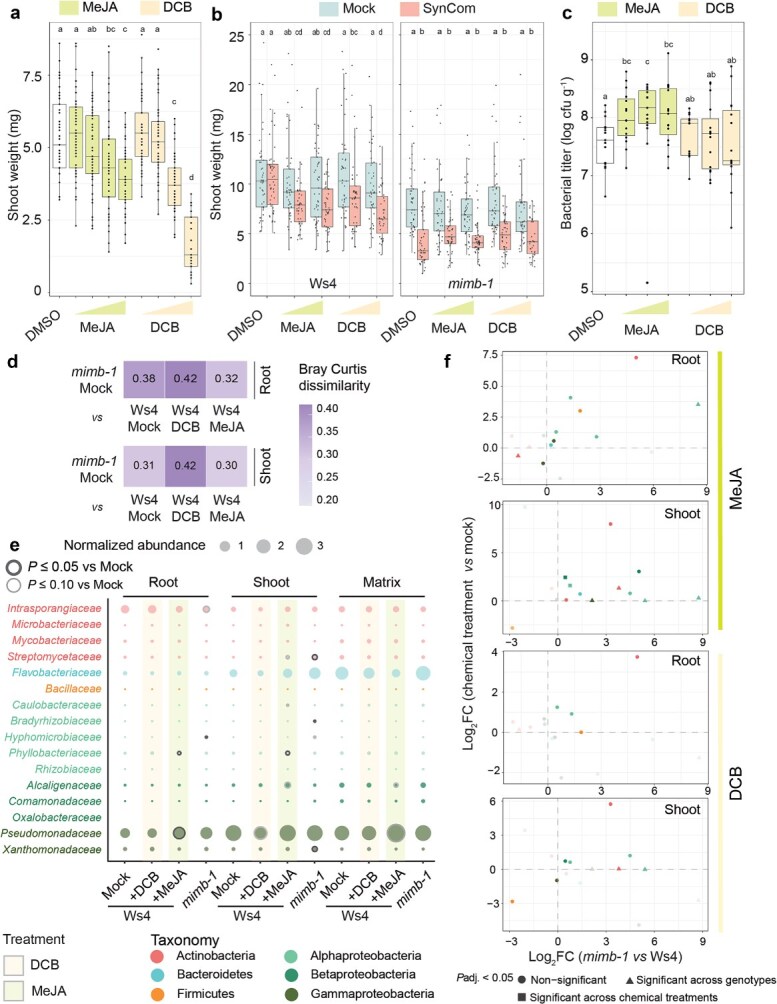
Treatments with two JA inducers partially reproduced *mimb-1* phenotypes on WT. (a) Effect of different concentrations of MeJA and DCB on plant growth. MeJA: 0.1, 0.25, 0.5, and 1.0 μM; DCB: 0.05, 0.1, 0.25, and 0.5 μM. Non-inhibitory concentrations of MeJA (0.1 and 0.25 μM) and DCB (0.05 and 0.1 μM) were used in the subsequent experiment with SynCom. (b) Treatment with different concentrations of MeJA or DCB induced SynCom-dependent growth inhibition on WT. MeJA: 0.1, 0.25 μM; DCB: 0.05, 0.1 μM. (c) Effect of different concentrations of MeJA or DCB on total microbial load on wild-type Ws4 plants. *At-*16SC2 was used in this experiment. MeJA: 0.5, 1, and 2.5 μM; DCB: 0.1, 0.25, and 0.5 μM. 0.1% DMSO was used as the mock control. (d) Heatmap summarizing the average Bray–Curtis dissimilarities between the indicated treatment. (e) Balloon plot summarizing the normalized abundance of different families colonizing the indicated compartments and genotypes after MeJA or DCB treatment. The same color code was used to represent microbial taxonomies. (f) Scatter plot showing the LFC of the abundance for individual strains between genotypes (x-axis, *mimb-1* mock vs Ws4 mock) and chemical treatment (y-axis, Ws4 MeJA or DCB treatment vs Ws4 mock). Each plot was divided into four quadrants with the upper right and lower left quadrants indicating strains (solid color) showing similar enrichment and depletion pattern in the respective comparison. Strains found in the upper left and lower right quadrants indicated strains (translucent colors) showing contrasting enrichment and depletion pattern. Colors represented taxonomies and shapes corresponded to statistical significance. Statistical significances were determined by ANOVA for (a) & (f), Kruskal*–*Wallis followed by Dunn’s *post hoc* test for (b) & (c), and pairwise Wilcoxon rank-sum test in (e). Different letters or symbols indicated statistical significance of *P* ≤ 0.05 unless otherwise specified.

If microbiota-mediated upregulation of JA indeed contributed to the microbial imbalance phenotypes, we expect that exogenous application of JA inducers in the presence of SynCom will phenocopy microbial imbalance and associated phenotypes on WT. To differentiate between direct chemical-mediated growth inhibition and growth modulation through interactions with the microbiota, we tested different concentrations of MeJA and DCB. DCB is a cell wall synthesis inhibitor known to induce JA signaling [71]. First, we identified the highest concentrations of MeJA and DCB not leading to significant plant growth reduction (Fig. 5a). Exogenous application of these two chemicals under non-inhibitory concentrations ranging from 0.05 to 0.25 μM together with the SynCom reproduced the growth inhibition phenotypes in WT plants (Fig. 5b), suggesting that the upregulation of the JA pathway resulted in synergistic microbiota-dependent growth inhibition. Unwounded and wounded Arabidopsis leaves were reported to accumulate between 5 and 150 ng JA g^−1^ fresh weight [72] (0.02–0.6 μM) so the concentrations we used were within physiological relevant ranges.

We investigated the impact of exogenous MeJA and DCB treatment on total microbial load. Using a similar setup, we showed that MeJA treatment led to five-fold higher microbial load on wild-type plants (Fig. 5c). Although DCB cotreatment with SynCom inhibited plant growth, variable and no statistically significant increase in total microbial load was observed. We also performed community profiling to investigate the impact of MeJA and DCB on community structure. The community composition of *mimb-1* differed from the first 16S rRNA sequencing result without chemical treatment, possibly due to the use of different growth chambers and batch effect variation. The community shift indued by MeJA and DCB were different and overlapped partially with those found in *mimb-1.* MeJA-treated but not DCB-treated wild-type plants showed higher similarity to *mimb-1* based on lower Bray–Curtis dissimilarity (Fig. 5d) and PERMANOVA analyses (Table S9, root, Ws4_DCB vs *mimb-1*_mock, *R*^2^ = 0.18, *P* = 0.05, Ws4_MeJA vs *mimb-1*_mock, *R*^2^ = 0.03, *P* = 0.74; shoot, Ws4_DCB vs *mimb-1*_mock, *R*^2^ = 0.21, *P* = 0.01, Ws4_MeJA vs *mimb-1*_mock, *R*^2^ = 0.07, *P* = 0.22). To better visualize the pattern of enrichment and depletion of individual strains across genotypes and chemical treatments, we performed differential abundance analysis (Fig. 5e and f). Using untreated Ws4 plants as the common reference, we calculated the LFC of individual strains upon chemical treatments or across genotypes. The resultant scatter plots showed that more strains were enriched or depleted with similar patterns as of *mimb-1* upon MeJA treatment compared to DCB (Fig. 5f). However, we did not find any strains showing significant enrichment or depletion under both MeJA treatment and *mimb-1* background, possibly suggesting partial mimicry between MeJA treatment and *mimb-1* genetic perturbation. We also noticed enrichment of *Pseudomonas* strains upon MeJA treatment in the agar matrix, suggesting that MeJA may affect the growth of some strains. We could not distinguish between primary and secondary effects upon upregulation of the JA pathway (e.g. plant morphological changes) on microbiota colonization because microbial overgrowth and community shift were coupled with varying levels of stunted plant growth.

To investigate whether some of the SynCom members may synthesize JA, thus contributing to the upregulation of the JA pathway, we performed genomic analyses using different SynCom members. Most of the members under investigation contained similar genes (>35% sequence identity) related to OPDA reductase and peroxisomal β-oxidation enzymes. However, none of them encoded the complete canonical JA biosynthesis pathway (Supp Fig. S6). Together, we provided evidence that plant microbiota composition and the plant JA signaling pathway was regulated by a microbiota-JA feedback mechanism, which was affected by yet identified genetic determinant(s) in the *mimb-1* mutant background.

### Misregulation of the jasmonate pathway in *mimb-1* altered plant responses to multiple stressors

**Figure 6:**
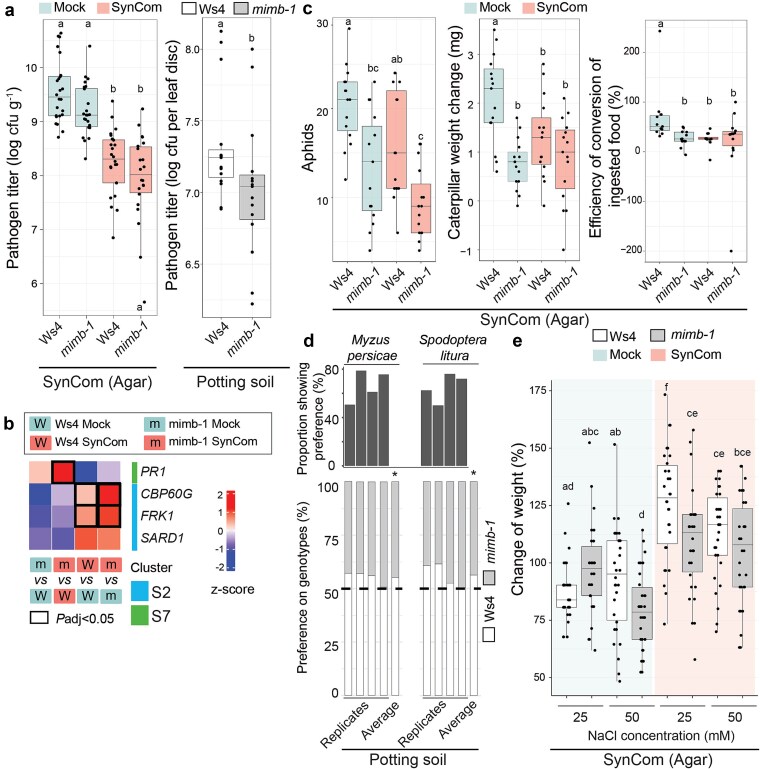
*mimb-1* mutant had altered responses to multiple stressors. (a) Bacterial load of the foliar bacterial pathogen *Pto*DC3000 on SynCom-inoculated (left) or soil-grown (right) WT and *mimb-1* plants. Bacterial load was quantified based on colony forming unit (cfu) per gram of tissue or per unit area of leaf disc. (b) Normalized relative expression levels of selected genes involved in SA-mediated defense responses in shoot. Genes of statistical significance (log_2_FC > 1.5, *P*adj. < 0.05) were indicated with blocked lines. (c) The relative fitness of aphids (fecundity) or caterpillars (weight gain and efficiency of ECI) upon feeding with Ws4 and *mimb-1* plants in non-choice experiments. (d) The preference of insects on Ws4 and *mimb-1* plants in free choice experiments (*n* = 4). The proportion in the upper panel corresponded to the percentage of insects exhibiting a preference on the respective genotypes. The lower panel corresponded to the selection rate on each genotype. (e) Sensitivity of Ws4 and *mimb-1* plants with an increasing concentration of salt. Sensitivity was expressed in terms of the percentage change of fresh weight compared to the mock treatment across both genotypes. Statistical significances were determined by Kruskal–Wallis followed by Dunn’s *post hoc* test for (a), (c) & (e), and beta regression test in (d). Different letters or symbols indicated statistical significance of *P* ≤ 0.05 unless otherwise specified.

To investigate the potential biological relevance of heightened JA signaling and microbial imbalance in association with stress adaptation, we performed multiple stressor experiments using both soil-grown plants and SynCom inoculated plants. Firstly, the role of microbiota in autoimmunity has been reported [30], we thus asked whether *mimb-1* exhibited heightened immunity level. To directly investigate whether *mimb-1* accommodated less pathogen load, we infected SynCom-inoculated or soil-grown *mimb-1* and WT plants with the hemibiotrophic pathogen *P. syringae pv. tomato PtoDC3000*. The results varied across these two experimental systems (Fig. 6a), possibly linked to the variation in environment and indigenous microbiota composition. In line with ecological theories including inter-microbial competition and niche occupation, pre-inoculation with SynCom led to significantly lower pathogen load upon infection regardless of the genotypes (Fig. 6a). No significant differences were observed between SynCom-treated *mimb-1* and WT (Fig. 6a). However, soil-grown *mimb-1* exhibited significantly lower pathogen load compared to WT (Fig. 6a). Autoimmune mutants were characterized by higher expression of defense marker genes (e.g. *SARD1, CBP60g, FRK1*, and *PR1)* [73]*.* The expression of *SARD1, CBP60g,* and *FRK1* were upregulated in response to SynCom across both genotypes in the shoot, whereas the expression of *PR1* was upregulated only in *mimb-1* (Fig. 6b,Table S10). Because JA and SA defense marker genes were upregulated, both JA-, SA-mediated immunity, and inter-microbial interactions contributed to reduced pathogen load.

Apart from resistance to bacterial pathogens, JA signaling also contributed to herbivore resistance. We performed non-choice tests by feeding sucking specialist aphid (i.e. *M. persicae)* and chewing generalist caterpillar (i.e. *S. litura)* with *mimb-1* or WT plants. As expected, aphids fed with *mimb-1* compared to WT resulted in significantly lower fecundity (Fig. 6c). Furthermore, aphids fed with SynCom-inoculated *mimb-1* led to significantly lower fecundity compared to SynCom-inoculated WT. In contrast, caterpillars fed with *mimb-1* compared to WT tended to exhibit lower weight gain and lower efficiency of ECI (Fig. 6c). However, no significant difference was observed between SynCom-inoculated *mimb-1* and WT (Fig. 6c), possibly reflecting different sensitivities against anti-herbivory defense between aphids and caterpillars. We also performed free choice test using soil-grown plants. Both insects exhibited significantly lower preference on *mimb-1* compared to WT (Fig. 6d).

We investigated the tolerance of *mimb-1* to an abiotic stressor such as high salinity. *mimb-1* mutant was hypersensitive to salt compared to WT under 50 mM salt treatment (Fig. 6e). However, the presence of SynCom did not affect the sensitivity across genotypes. Together, these results indicated that the JA hyperactive *mimb-1* mutant had altered resistance and tolerance to multiple stressors.

## Discussion

### Identification of *FLAG_264G01* as a mutant with microbial imbalance

Over the last decade, multiple factors implicated in plant microbiota establishment have been identified [1, 3, 4, 8, 18, 28, 74, 75]. In the case of root microbiota, edaphic factors play deterministic roles by shaping the composition of the initial soil inoculum for subsequent root colonization. Cell wall features, root exudation linked with microbial resources preferences, and plant innate immunity work together to fine-tune microbiota composition. Despite these advances, the genetic basis underlying plant microbiota homeostasis is not fully explored. Several dysbiosis mutants were reported to share a common or suspected role in plant innate immunity [23–26, 30, 76]. Here we reported a mutant with microbial imbalance phenotypes (*microbial imbalance-1* or *mimb-1*). Despite extensive effort, we are still in the process confirming the genetic determinant(s) contributing to microbial imbalance in the *mimb-1* background. Whole genome sequencing will help to identify unknown candidate(s) for validation. Despite a lack of genetic information, *mimb-1* exhibited “bona fide” features of dysbiosis and was unique in several ways. First, *mimb-1* derived microbiota failed to reproduce the microbial imbalance phenotypes on WT, suggesting a mechanism to restore the otherwise mutant-like community back to normal. Second, the associated microbial overproliferation was not specific to a single bacterial family. Third, *mimb-1* resembled autoimmune mutants such as *snc1* [77] and *tip1* [30] with a stunted growth phenotype. However, *snc1* and *tip1* exhibited growth defects under axenic conditions and microbiota alleviated this phenotype, whereas microbiota-mediated stunted growth was aggravated in *mimb-1* (Fig. 1a). Elicitor hypersensitivity was unlikely as treatment of *mimb-1* with the heat-killed SynCom, presumably carrying a cocktail of heat-stable elicitors, did not result in stunting (Fig. 1b). The lack of clear evidence linking microbial imbalance to altered immunity is in line with the observation that both immune activating and suppressing SynCom resulted in microbial imbalance. Based on our transcriptomic and JA quantification assays (Figs 3–4), we then moved our attention to the upregulated jasmonate signaling pathway as the mechanistic cause of microbial imbalance.

### Upregulation of the jasmonic acid pathway contributed to altered plant microbiota interactions

The role of JA in shaping microbiota composition has been investigated, but their conclusions varied [33–36, 38]. Such discrepancies were likely compounded by variations in plant species, soil parameters, microbiota composition, and the method of community analyses (e.g. sequencing versus PhyloChip). Additionally, volatiles such as MeJA acted directly on microbes by affecting biofilm formation [78]. JA-mediated alternations in root exudation profile were correlated to community shift [79], suggesting that JA may affect microbiome through the regulation of root exudate chemistry. To investigate the possibility of a feedback loop between JA and the bacterial microbiota, we performed reconstitution experiment. Colonization by SynCom resulted in higher levels of JA, which was amplified in the *mimb-1* mutant background. Conversely, treatment of JA inducer partially phenocopied *mimb-1* including microbiota-dependent stunted growth, microbial overgrowth, and community shift (Fig. 5), suggesting that the upregulation of JA signaling directly altered plant–microbiota interactions. We speculate that this JA-dependent feedback loop functions within physiological relevant ranges in WT. However, due to unidentified genetic perturbation in the *mimb-1* mutant, this feedback loop was amplified. The upregulation of the JA pathway compromised plant growth and impacted plant responses to both abiotic and biotic stressors (Fig. 6). Together, our results underscore the importance of precise hormonal regulation to sustain healthy plant–microbiota interactions.

### Interplay between plant microbiota and stress adaptation through a jasmonic acid-feedback loop

Upregulation of JA signaling is essential for plant growth and adaptation to environmental stresses. For instance, upregulation of the JA pathway can promote plant disease susceptibility through the suppression of the SA branch, a virulence strategy deployed by some pathogenic strains to promote infection [80, 81]. However, *mimb-1* mutant was more resistant to *Pto*DC3000, at least in terms of pathogen load. Enhanced pathogen resistance associated with higher JA signaling was reported in the *cev* mutant [82, 83], corresponding to a mutation in the cellulose synthase *CeSA3* [71]. Unfortunately, we did not get *cev* to investigate the general role of JA upregulation for our phenotypes. Instead, we used two different JA inducers. Treatment of plants with the JA inducer, DCB, had a strong growth inhibitory effect but insignificant effect on microbial load (Fig. 5c), possibly suggesting specific JA-mediated branch (e.g. through different JAZs) contributing to microbial imbalance. It will be instrumental to generate mutants by CRISPR editing or crossing *mimb-1* with a JA mutant to test this hypothesis. However, it requires JA mutants in the same genetic background as *mimb-1* and prior knowledge of the identity of the underlying genetic determinants to minimize failure of segregation due to genetic linkage.

Defect in cell wall integrity activated plant innate immunity [84]. It is possible that the reduced pathogen load of *Pto*DC3000 of *mimb-1* was a result of both altered inter–microbial interactions and immune response. Additionally, community shift contributed to pathogen resistance through the activation of ISR [37, 85–87]. In contrast, upregulation of the JA pathways enhanced plant resistance to herbivores, which was in line with our herbivore non-choice experiments (Fig. 6C and D). However, *mimb-1* mediated defense performed better on aphids than caterpillars and not necessarily depended on SynCom colonization. A possible explanation was that caterpillars suppressed JA-mediated phloem defense through oral secretions [88], contributing to the differential susceptibilities between aphids and caterpillars [89, 90]. Alternately, *mimb-1* may mount resistance through JA-independent processes. For instance, plant accumulated toxic phenolic precursors to fend off herbivores [91]. Another phytohormone, SA, was also induced during plant–aphid interactions [92], especially upon incompatible but not compatible interactions [93]. These studies suggested that SA also played a role in resistance against aphids. Provided that SA and JA have known cross-talk and some SA marker genes were upregulated in *mimb-1,* we cannot exclude the contribution of both enhanced JA- and SA-mediated defense against herbivores. Together, our results highlighted the complexity underlying JA-dependent and independent defense and the corresponding differential sensitivities by the insect herbivores.

Upregulation of JA signaling can confer abiotic stress tolerance. For example, JA was synthesized in response to high salinity and resulted in the degradation of JAZ repressor proteins through the COI1-dependent proteasomal degradation pathway. Degradation of JAZ8 led to the derepression of the salt-responsive NF-YA1-YB2-YC9 complex [94], thereby contributing to salt tolerance. Enhanced level of JA was associated with the degradation of JAZs [32]. However, in the case of *mimb-1*, enhanced JA level was coupled with the transcriptional upregulation of multiple *JAZs*, including *JAZ8* in the shoot (Supp Fig. S4). Upregulation of JAZs may account for the reduced salt stress tolerance in *mimb-1* mutants despite an overall upregulation of the JA signaling pathway. Together, these results highlight potential crosstalk of the underlying regulatory network. Interactions with the microbiota should be considered, with the overarching goal of promoting both plant growth and stress tolerance.

## Supplementary Material

Supplementary_material_wrag164

## Data Availability

RNA-seq (Accession: PRJNA1314934) and 16S rRNA gene sequencing data (BioProject: PRJNA1321540) were uploaded in the public domain. JA and SA quantification data were uploaded to massive.ucsd.edu, and can be assessed through the Identifier MSV000098655. Scripts to reproduce our RNA-seq and 16S rRNA gene sequencing data can be found in the Github link https://github.com/migzisip/PER5_Microbiota_JA_signaling.
