# Supplementary material for: Upregulated jasmonate signaling shifts Arabidopsis microbiota interactions and stress adaptations through a positive feedback loop
Source: ISME J. 2026 Jun 11;20(1):wrag146. doi: 10.1093/ismejo/wrag146 (PMC13352530; doi:10.1093/ismejo/wrag146)
Supplement: Supplementary_material_wrag164 [file supplementary_material_wrag164.zip › Supp_Figure_S1-S6_Final_wrag146.docx]

**
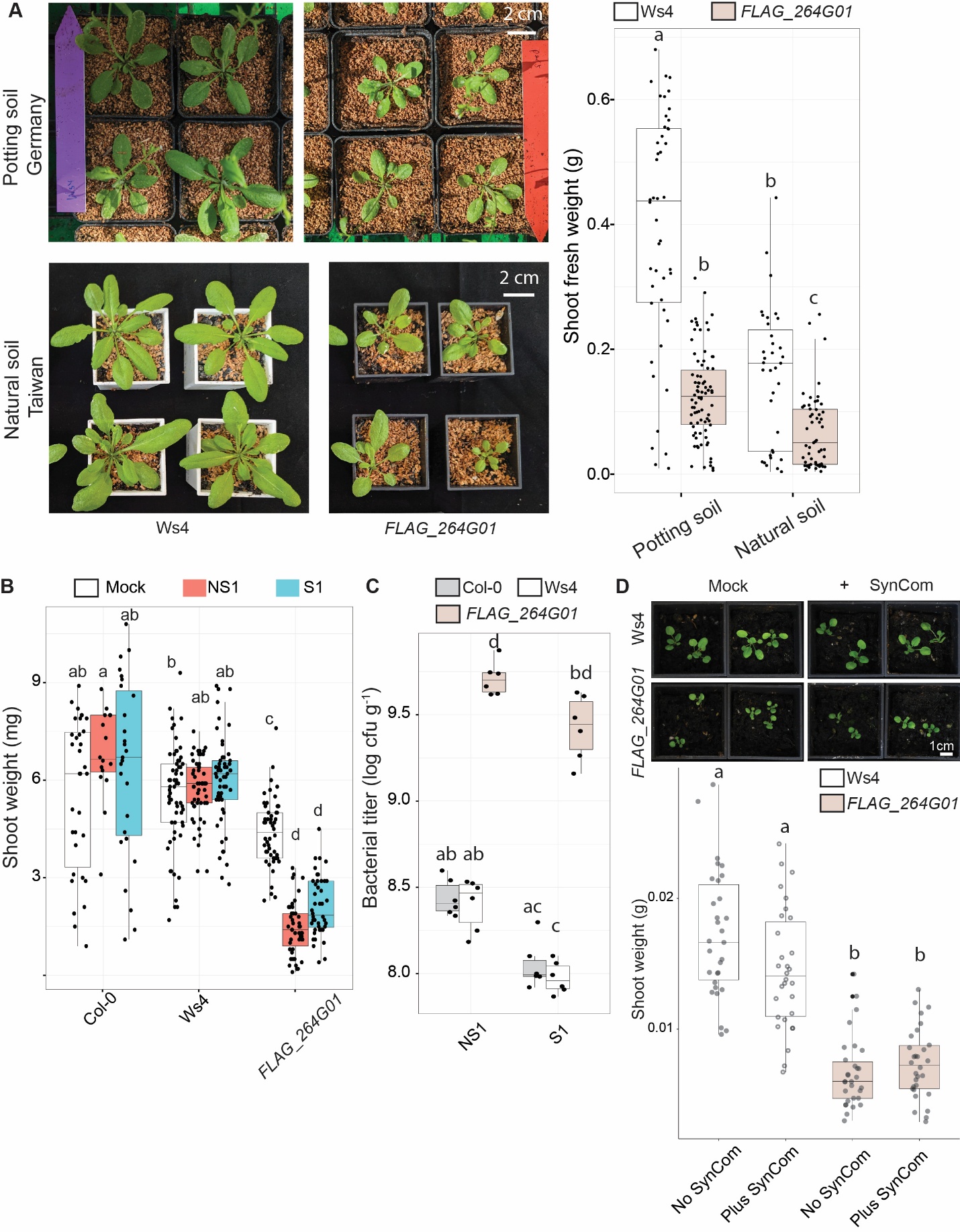
**

**Supp S1.** **Growth inhibition and microbial overgrowth were reproducible in *FLAG_264G01* using different synthetic communities**. (A) Representative images of 5-week old Ws4 and *FLAG_264G01* grown in peat-based potting soil and natural soil. Experiments were performed separately in two greenhouses in Germany and Taiwan. Shoot fresh weights of plants grown in both potting soil and natural soil collected from Taiwan. The experiment was repeated a total of two times. (B) & (C) Shoot fresh weight and total microbial load of two-week old plants inoculated with the 5-member immune non-suppressive (NS1) and suppressive SynCom (S1). (D) Shoot fresh weight of two-week old soil-grown plants with weekly treatment of SynCom *At*-16SC2. The experiment was performed once. Statistical significance was determined by Kruskal-Wallis followed by Dunn’s post-hoc test. Different letters indicated statistical significance of *P*≤0.05 unless otherwise specified.

**
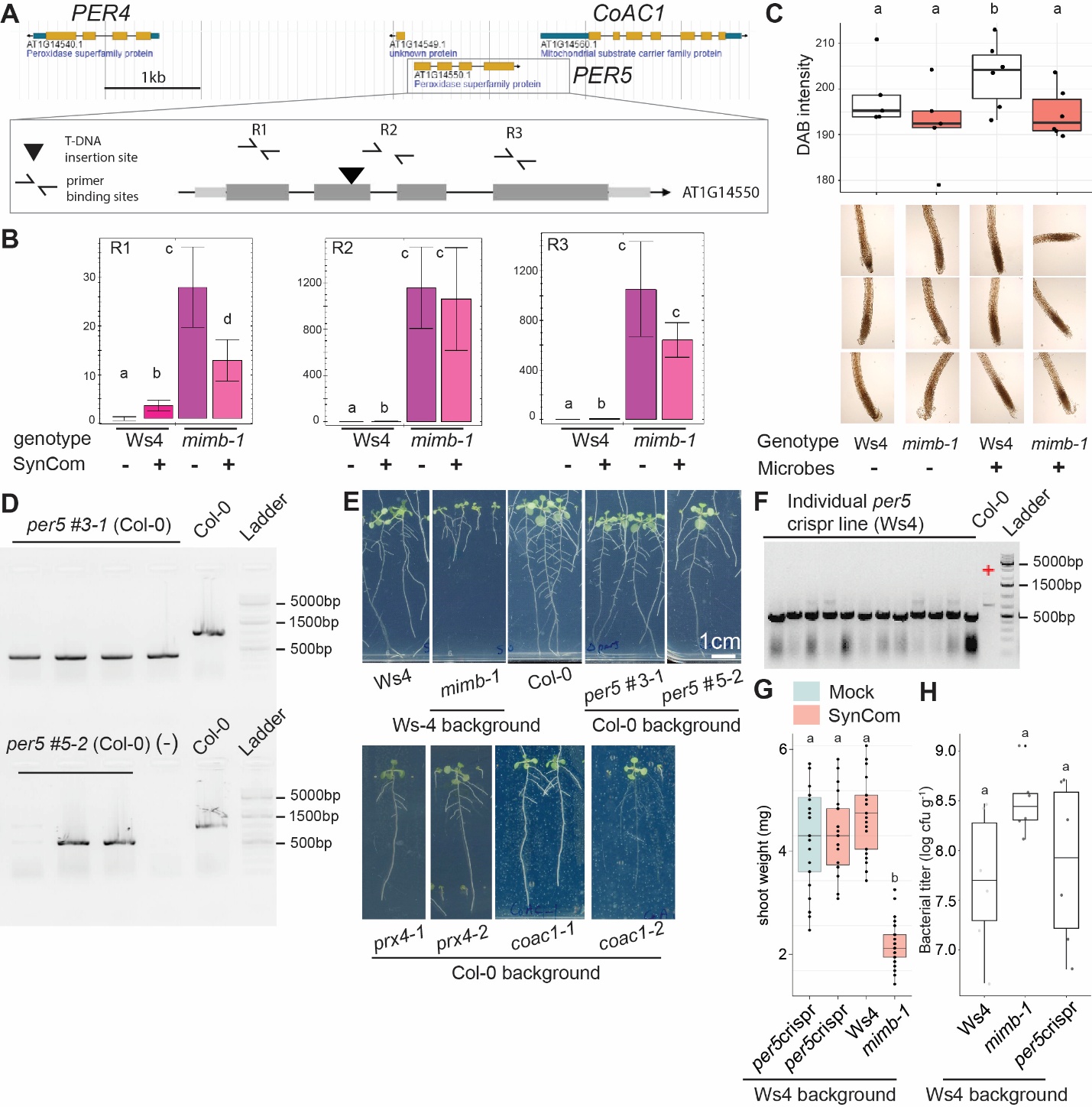
**

**Supp S2. Mutation of the *PER5* gene is not sufficient to cause dysbiosis phenotypes**. (A) Schematic diagram showing the coding region of *PER5* (*AT1G14550*) and the surrounding 2kb windows upstream of the start codon and downstream of the stop codon of *PER5*. A T-DNA (indicated by a block triangle) is inserted into the second exon of *PER5* in the mutant line *FLAG_264G01*. (B) The expressions of transcripts upstream and downstream of the T-DNA insertion site as quantified by quantitative RT-PCR. (C) Intensity of DAB as a proxy of the level of reactive oxygen species (ROS) under mock treatment or bacterial inoculation. Plants were inoculated with the *Pseudomonas* strain R9. The experiment was repeated a total of two times. (D) Confirmation of deletions in two independent *PER5* CRISPR lines generated in the Col-0 background by PCR. Accession Col-0 was included as a control. (E) Representative images of *PER5* deletion mutants (*per5 #line 3-1 and 5-2*), *PER4* knock-down mutant (SALK_110617C, *prx4-1*), *PER4* hypermorphic mutant (SALK_044730C, *prx4-2*) and *COAC1* null mutants (SALK_091671C, *coac1-1*; SALK_087365C, *coac1-2*) two weeks after inoculation with the 16-member SynCom. The experiment was repeated a total of two times. (F) Confirmation of deletions in individuals of a *PER5* CRISPR deletion line generated in the Ws-4 background by PCR. Accession Col-0 was included as a control (indicated with a cross). Both Col-0 and Ws4 give rise to a *PER5* amplicon of the same size. (G) & (H) Shoot fresh weight and total microbial load of two-week old plants inoculated with the SynCom *At*-16SC2. The experiment was performed once. Statistical significance was determined by Kruskal-Wallis. Different letters indicated statistical significance of *P*≤0.05.


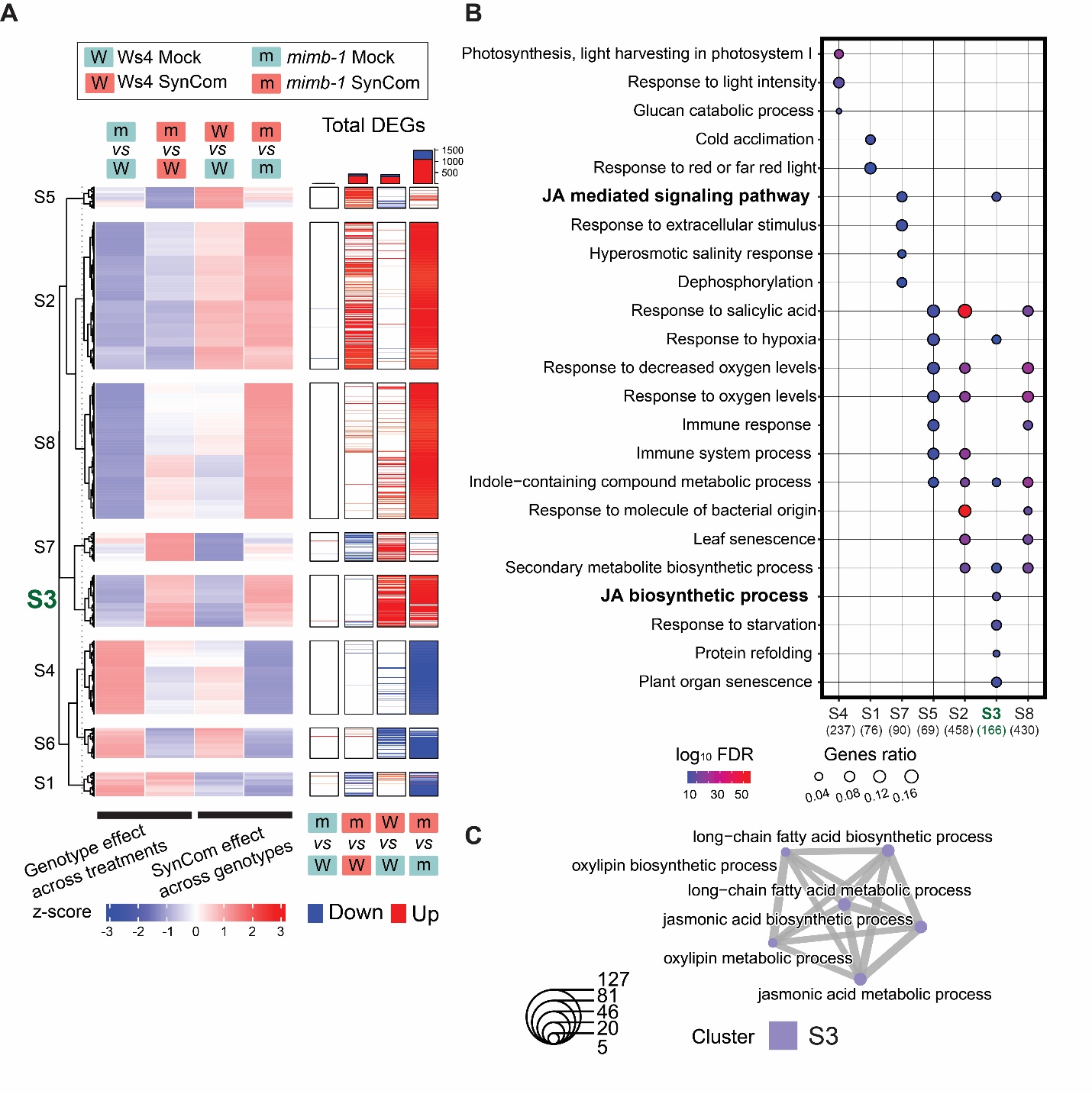


**Supp S3. Shoot transcriptomes of WT and *mimb-1* plants in response to SynCom.** (A) Heatmaps summarizing the relative expression of genes across genotypes in shoots. DEGs were calculated by pairwise comparisons (log_2_FC>1.5, *P*<0.05). (B) Top five significantly enriched GO terms associated with each cluster for the shoot dataset. (C) GO terms related to JA processes and their association with clusters S3. Size of pie charts corresponded to the number of DEGs.


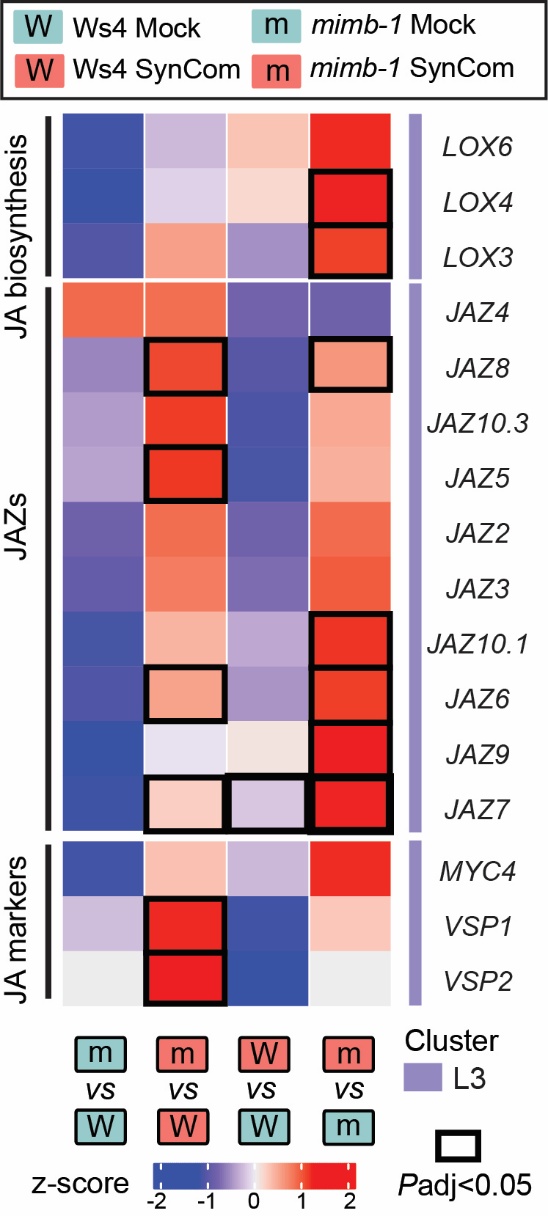


**Supp S4. JA related genes were upregulated in *mimb-1* in response to the synthetic community in shoot**. Normalized relative expression levels of selected genes involved in JA-related processes in shoots (related to Fig 3B). Genes of statistical significance (log_2_FC>1.5, *P*<0.05) were indicated with blocked lines.

**
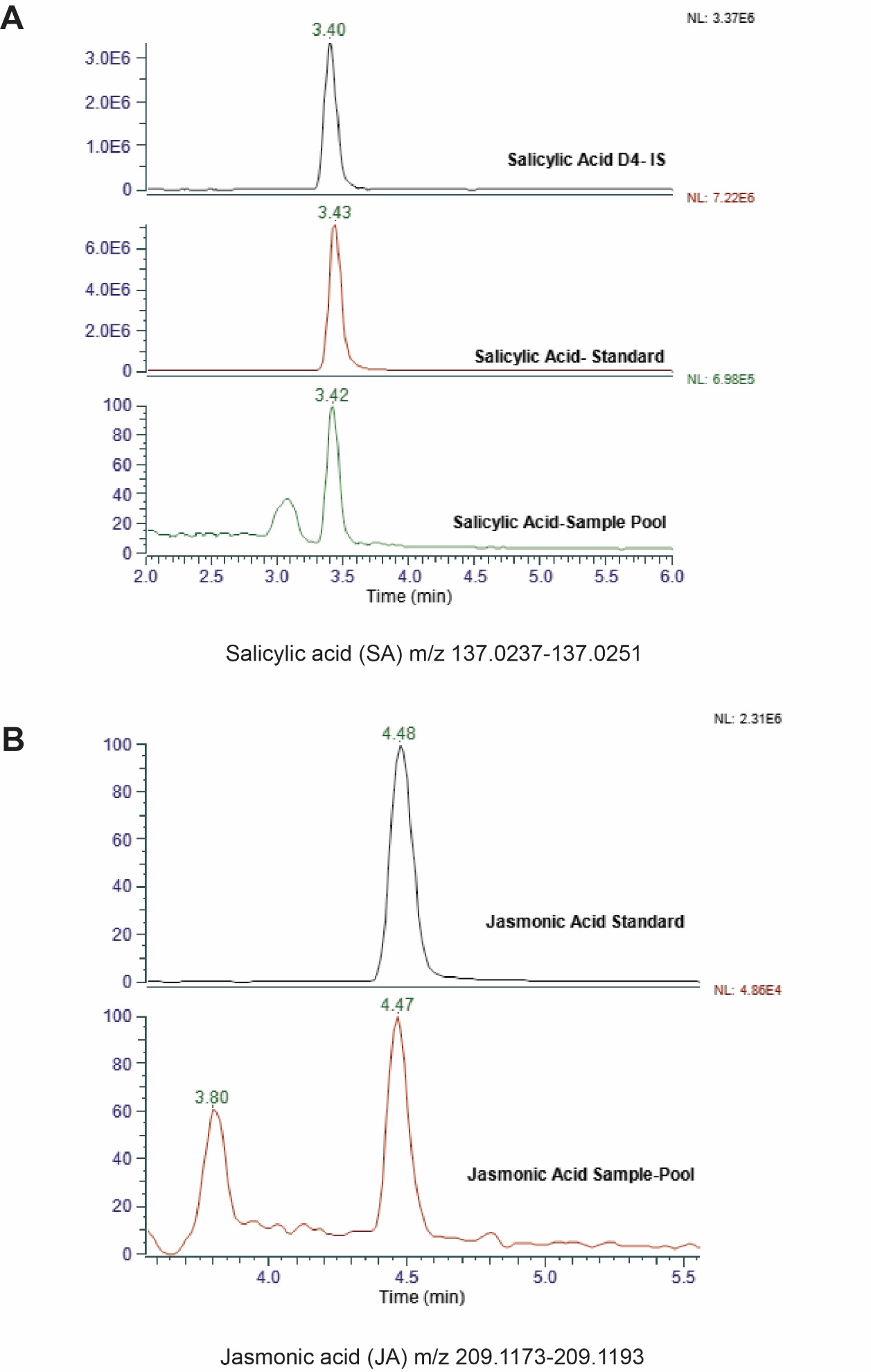
**

**Supp S5. Quantification of JA and SA for WT and *mimb-1* plants.** Overlay chromatograms of JA and SA with their corresponding internal standards.


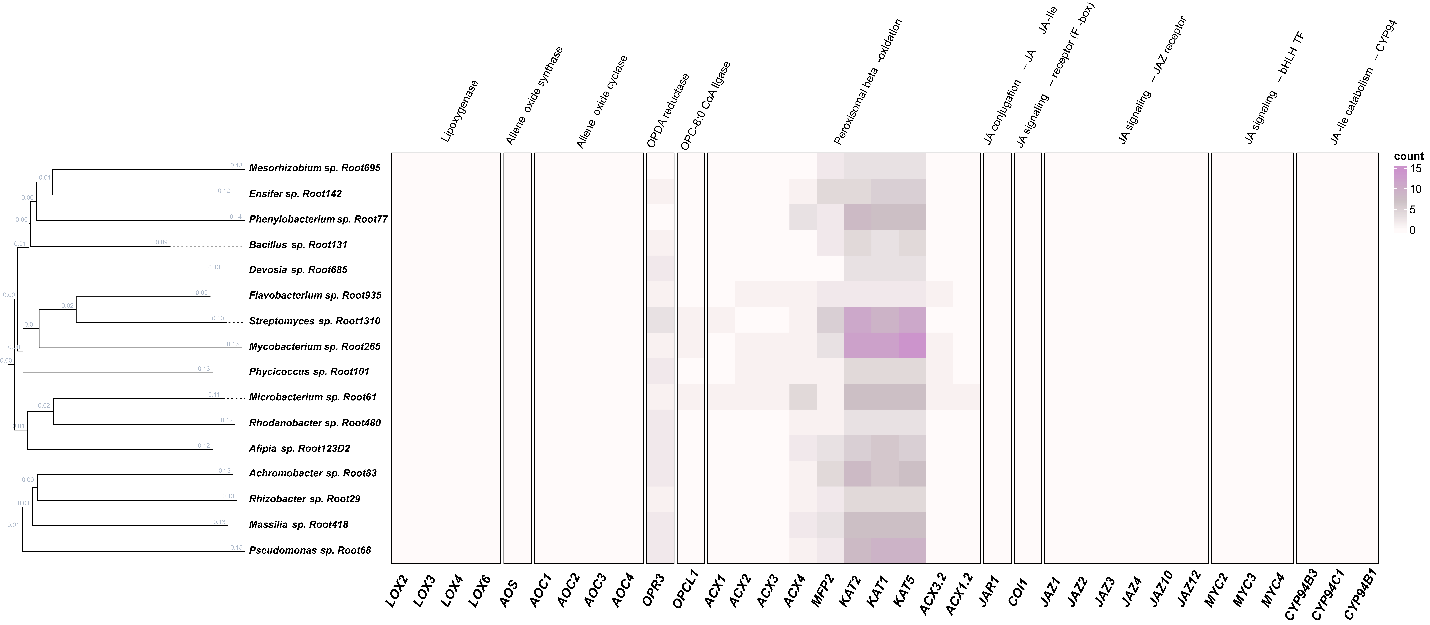


**Supp. S6.** **Genomic analyses for JA metabolic genes in different commensal bacteria**. Phylogenetic tree showing the presence of specific genes related to JA metabolic pathway using the homologs from *Arabidopsis thaliana* as a reference. Genes showing more than 35% sequence identity to the reference were kept in our analyses. The percentage of identify was visualized as a heatmap.
